# Extracellular Vesicle-Associated Angiopoietin-2 and Cell Migration-Inducing Protein in Lung Cancer Progression and Brain Metastases

**DOI:** 10.7759/cureus.80200

**Published:** 2025-03-07

**Authors:** Flaviu Tamas, Corina I Tamas, Bogdan A Suciu, Adrian F Balasa

**Affiliations:** 1 Neurosurgery, Doctoral School of Medicine and Pharmacy, “George Emil Palade” University of Medicine, Pharmacy, Science and Technology, Târgu Mureș, ROU; 2 Neurosurgery, Emergency Clinical County Hospital, Târgu Mureș, ROU; 3 Thoracic Surgery, Emergency Clinical County Hospital, Târgu Mureș, ROU

**Keywords:** angiopoietin-2, brain metastasis, cell migration-inducing protein, extracellular vesicle, lung cancer

## Abstract

Background: Angiopoietin-2 (ANGPT2) and cell migration-inducing protein (CEMIP) are key regulators of angiogenesis, extracellular matrix remodeling, and metastatic progression in various cancers, including lung cancer (LC). The presence of these biomarkers in extracellular vesicles (EVs) may offer valuable insights into the molecular mechanisms underlying LC progression and metastasis. Extracellular vesicles play a critical role in LC by enhancing intercellular communication and supporting processes such as angiogenesis, immune evasion, and metastasis, transferring key molecules like vascular endothelial growth factor (VEGF) and pro-angiogenic microRNAs (miRNAs).

Methods: This study aimed to investigate the presence and quantification of ANGPT2 and CEMIP in the cargo of EVs isolated from plasma samples obtained from the peripheral venous blood of patients with localized lung cancer (LLC group), lung cancer with brain metastases (LCM group), and healthy controls (HC group). EVs were isolated using the density gradient ultracentrifugation method, and their characterization was performed through scanning and transmission electron microscopy as well as flow cytometry. Western blot analysis was used to identify ANGPT2 and CEMIP in EV cargo, and band intensity from western blot images was quantified using specialized software.

Results: The expression of ANGPT2 and CEMIP in EV cargo was significantly higher in the oncologic groups (LLC and LCM combined) compared to the HC group. Notably, EV CEMIP levels were, on average, 59% higher in patients with brain metastases than in those with localized lung cancer. Following surgical resection, postoperative EV ANGPT2 and EV CEMIP levels decreased by 36% and 8.5%, respectively, in the LLC group, and by 40% and 4.6%, respectively, in the LCM group.

Conclusion: These findings emphasize the potential of ANGPT2 and CEMIP as biomarkers for LC progression and prognosis. To our knowledge, no previous study has evaluated the presence and quantification of ANGPT2 and CEMIP in EV cargo from lung cancer patients. To further validate their role in cancer progression, functional studies should explore the mechanistic effects of EV-associated ANGPT2 and CEMIP on angiogenesis, immune modulation, cell migration, extracellular matrix remodeling, and tumor progression in lung cancer models.

## Introduction

Lung cancer (LC) ranks first among the causes of cancer-related deaths worldwide, resulting in more deaths than breast, colon, and prostate cancers together [1]. Histologically, LC is divided into small cell lung cancer (SCLC), representing 15% of LC, and non-small cell lung cancer (NSCLC), which represents 85% of LC. NSCLC is histologically subdivided into two primary subtypes: lung adenocarcinoma and lung squamous cell carcinoma [2,3].

Recent research has highlighted the significance of exosomes in cancer development, particularly their involvement in organotropic metastatic spreading [4-9]. These lipid-bilayer extracellular vesicles (EVs), 20-100 nm in diameter, enhance intercellular communication by transferring proteins, lipids, and genetic material, influencing the extracellular matrix and supporting metastasis [5]. Cancer cells secrete exosomes in greater quantities than normal cells, with altered content promoting tumor growth, immune evasion, angiogenesis, and premetastatic niche formation [6]. Exosomes also contribute to chemotherapy resistance by transferring drug-resistance proteins between cancer cells [7]. Isolated from various body fluids, exosomes are typically purified through ultracentrifugation and density gradient methods for high specificity [8]. Exosomes hold significant potential as targets for early cancer detection, prognosis assessment, therapy, and monitoring treatment responses [9]. Their application as liquid biopsies, particularly for diagnosing central nervous system metastases using blood or cerebrospinal fluid samples, is promising, especially for deep-seated lesions where traditional biopsies pose risks or when distinguishing from primary gliomas. Beyond diagnosis, EVs also offer opportunities for molecular tumor profiling [10]. Current studies concentrate on utilizing EVs as drug delivery vehicles for crossing the blood-brain barrier, offering targeted delivery with reduced toxicity [11,12]. Techniques like focused ultrasound (FUS) with microbubbles (MBs) may further improve their therapeutic potential for brain diseases [13].

LC development is linked to the activation of oncogenes, inactivation of tumor suppressor genes, protein functional effector small non-coding ribonucleic acid (sncRNAs), angiogenesis, and inflammation [14]. EVs serve an essential role in lung carcinogenesis by delivering nucleic acids and proteins to specific cells [15]. Angiogenesis, crucial for tumor progression, involves endothelial proliferation and organization, aiding tumor growth, resistance to apoptosis, and metastasis [16]. Key regulators include angiopoietins (ANGPT), the Tie2 receptor, and vascular endothelial growth factor (VEGF) pathways, which influence vascular stability and maturation [17]. Angiopoietin-2 (ANGPT2) is important in tumor angiogenesis, destabilizing blood vessels through competitive binding to the Tie2 receptor of endothelial cells [18]. Studies have revealed that ANGPT2 may have a double function in angiogenesis, acting predominantly as a Tie2 antagonist essential for neovascularization in cancers or as a weak Tie2 agonist, which is essential for lymphatic vessel development [19,20]. This destabilization facilitates new blood vessel formation in the presence of VEGF, promoting tumor growth. In lung cancer, ANGPT2 is preferentially expressed and acts as an "angiogenic switch," transitioning tumors from a non-vascularized to a vascularized state. Under hypoxic conditions, ANGPT2, along with VEGF, drives endothelial cell migration, proliferation, and vessel sprouting [20]. While serum ANGPT2 shows potential as a marker for LC progression, its correlation with patient prognosis remains unclear due to conflicting study results [21]. Although ANGPT2 is an important factor in LC progression and metastasis, it operates within a complex network of other critical factors. Studies have highlighted that ANGPT2 is not only essential for cancer cell angiogenesis but also serves as an indicator of poor prognosis, metastasis, and invasion [22]. However, other factors like vascular endothelial growth factor A (VEGFA) also play crucial roles. For instance, both ANGPT2 and VEGFA protein levels are strongly associated with tumor dimensions and metastasis in lymphatic nodes in lung adenocarcinoma but not in squamous cell carcinoma. Elevated levels of these proteins correlate with poorer overall survival in adenocarcinoma patients [23]. Therefore, while ANGPT2 is important, it is one of several factors contributing to lung cancer progression and metastasis.

Cell migration-inducing protein (CEMIP), also known as KIAA1199, is a protein encoded by the CEMIP gene. It has an important role in cell migration, extracellular matrix remodeling, and cancer progression, particularly in LC. CEMIP has a role in hyaluronan degradation, influencing cell adhesion and motility, which facilitates tumor invasion and metastasis. Its overexpression has been linked to angiogenesis, inflammation, and immune modulation, creating a pro-tumor microenvironment that promotes cancer progression [24,25]. In NSCLC, high levels of CEMIP are associated with enhanced epithelial-mesenchymal transition (EMT), increased metastatic potential, and poor prognosis, particularly in cases of brain metastases. Additionally, CEMIP contributes to tumor cell survival under hypoxic conditions and has been implicated in chemoresistance, making it a potential therapeutic target [26]. Given its significant role in lung cancer, further research into CEMIP inhibition and its effect on tumor development may provide valuable insights for developing novel treatment strategies.

The aim of this study was to determine whether ANGPT2 and CEMIP are present in the EV cargo isolated from the peripheral blood of LC patients and to evaluate whether the quantity of EV ANGPT2 and CEMIP differs among patients with localized lung cancer without metastases, those with lung cancer and brain metastases, and non-cancerous individuals. Additionally, we sought to investigate how ablative surgical intervention influences the EV ANGPT2 and EV CEMIP of cancer patients. To assess the inflammatory status of the enrolled patients, we also calculated the average value of the systemic immune inflammation index (SII) for each group of patients.

## Materials and methods

Patient recruitment

This 20-month prospective observational study (January 2023 to September 2024) was carried out in the Neurosurgery and General Surgery Departments of the Emergency Clinical County Hospital of Târgu Mureș, Romania, following approval from the Local Ethics Committee (decision no. 30292/08.12.2020). Experimental analyses were conducted at the Immunology Laboratory of the Advanced Medical and Pharmaceutical Research Center, affiliated with the "George Emil Palade" University of Medicine, Pharmacy, Science, and Technology in Târgu Mureș, Romania. All procedures complied with the ethical principles of the Declaration of Helsinki for research involving human participants. Written informed consent was obtained from all individuals included in the study.

Patients were divided into three groups. The first group consisted of 11 patients diagnosed with localized lung cancer without metastases (LLC group), who underwent surgical treatment in the Thoracic Surgery Unit of the General Surgery Department. The second group consisted of 13 patients with lung cancer and brain metastases (LCM group) who underwent surgery in the Neurosurgery Department and were included only if a gross-total resection of all brain metastases was achievable. The third group was the control group, which comprised 12 healthy subjects (the HC group).

The inclusion criteria for patients in the LLC group required a histologically confirmed diagnosis of lung cancer, with non-metastatic bronchopulmonary cancer according to the eighth edition of the tumor, node, metastasis (TNM) classification (stages M0, T1-T4, N0-N3) [27], no prior oncologic treatment, absence of active infections, and a functional status with a Karnofsky performance score (KPS) of at least 80.

The inclusion criteria for patients in the LCM group required histopathological confirmation of bronchopulmonary cancer with brain metastases, classified as M1b or M1c according to the eighth edition of the TNM classification [27], based on the presence of a single or multiple metastatic lesions, no prior oncologic treatment except for corticosteroids used to alleviate symptoms of increased intracranial pressure, absence of active infections, and a functional status with a KPS of at least 80.

The inclusion criteria for the healthy control group required age, sex, and smoking history matching with the cancer patient groups, absence of any history of cancer, chronic neurological disorders, autoimmune diseases, or other significant medical conditions, as well as no prior oncologic treatment, including chemotherapy or radiotherapy.

Each patient in the LLC and LCM groups underwent comprehensive evaluations, including whole-body contrast-enhanced computer tomography (CT) scans. Additionally, cranial magnetic resonance imaging (MRI) was performed for patients in the LCM group. For the patients included in the HC group, low-dose CT scans excluded cancer. Each patient underwent routine laboratory investigations as part of the evaluation. Data was compiled from the Hospital Information System and supplemented with information provided directly by the patients or their legal representatives. This dataset included variables such as gender, age at diagnosis, tumor imaging characteristics (e.g., location and size, measured as the largest axial diameter on contrast-enhanced MRI or CT scans in millimeters; for patients presenting with multiple metastases, we measured and included in the study the diameter of the largest metastasis), histopathological features, and clinical status assessed using KPS.

Exclusion criteria for the LLC group were the following: patients with metastatic disease classified as M1a, M1b, or M1c according to the eighth edition of the TNM classification [27], lack of histopathological or cytological confirmation of the diagnosis of lung cancer, a KPS below 80, prior oncologic treatment (chemotherapy, radiotherapy, or targeted therapy), active or uncontrolled infections, coexisting malignancies or a history of another primary cancer within the last five years (except for non-melanoma skin cancer), severe comorbid conditions that could interfere with study outcomes (such as advanced cardiac, hepatic, or renal failure), neurological disorders unrelated to brain metastases that could impair functional assessment, major surgical procedures within the six months preceding study inclusion and patients unable or unwilling to provide informed consent.

Exclusion criteria for the LCM group were the following: patients with multiple primary malignancies, lack of histopathological or cytological confirmation of the diagnosis of lung cancer, prior oncologic treatment (chemotherapy, radiotherapy, immunotherapy or targeted therapy) except for corticosteroids used to manage symptoms of raised intracranial pressure, presence of active or uncontrolled infections, severe systemic comorbidities that could interfere with prognosis (such as advanced cardiac, hepatic, or renal failure), uncontrolled intracranial hypertension or brain edema, neurological conditions unrelated to brain metastases that could impair functional assessment, a KPS below 80, major surgical procedures within the six months preceding study inclusion or patients unable or unwilling to provide informed consent.

Exclusion criteria for the healthy control group were the following: any history of cancer or significant medical conditions that could influence study outcomes, severe or uncontrolled chronic diseases, recent major surgery or hospitalization within the past six months, use of medications that could affect study parameters, such as immunosuppressive drugs, a history of excessive alcohol consumption or drug abuse, abnormal imaging findings, and inability to understand the study procedures and provide informed consent.

Following surgery, all patients underwent postoperative oncological treatment tailored to their histopathological classification.

Peripheral blood sample collection

Participants in the first two groups were evaluated at two critical time points: preoperatively and seven days postoperatively. For the control group, a single peripheral venous blood sample was collected from each participant.

Peripheral blood samples were obtained after overnight fasting using 9-mL K2-ethylenediaminetetraacetic acid (EDTA) vacutainers (Becton Dickinson, Franklin Lakes, NJ, USA). Samples were processed in the first two hours using a two-step centrifugation protocol: an initial centrifugation at 300 × g for 10 minutes at 4°C for removing intact cells, followed by a second centrifugation at 2000 × g for 20 minutes at 4°C for removing platelets, cellular debris, and apoptotic bodies. The resulting plasma was stored at -80°C until all samples were collected.

Extracellular vesicle isolation from plasma samples

Plasma processing and EV isolation were carried out following the guidelines specified in the latest edition of the Minimal Information for Studies of Extracellular Vesicles (MISEV2023), updated in 2023 [28].

Plasma samples were briefly thawed, and EVs were isolated using the density gradient ultracentrifugation (DGU) method, performed with a CP100NX ultracentrifuge fitted with a Hitachi P23ST rotor (Hitachi High-Tech Science Corporation, Tokyo, Japan). The density gradient was prepared by creating serial dilutions of iodixanol (OptiPrep^TM^, Axis-Shield PoC AS, Oslo, Norway) in 30 mM Tris-HCl buffer (pH 7.5) supplemented with 0.25 M sucrose. This process resulted in four distinct layers with varying concentrations and densities: 40% (1.255 g/cm³), 20% (1.150 g/cm³), 10% (1.097 g/cm³), and 5% (1.065 g/cm³). Each patient's 1.4 ml of thawed plasma was carefully layered on top of the four density gradient layers and subjected to ultracentrifugation at 24,000 rpm for 18 hours at 4°C. During this process, EVs, including microvesicles, apoptotic bodies, and exosomes, were separated based on their respective densities. After separation by DGU, a second ultracentrifugation was carried out at 24,000 rpm for one hour at 4°C to enhance the purity of the suspension.

Extracellular vesicle characterization by scanning and transmission electron microscopy (STEM)

The size distribution, shape, and bilayer membrane of EVs isolated by DGU were analyzed using STEM. EVs stored at -80°C were quickly thawed and combined with an equal amount of glutaraldehyde, resulting in a final concentration of 2.5%. EVs were applied to Formvar-carbon-coated grids. The EVs were visualized and imaged using a Hitachi HD-2700 scanning transmission electron microscope (STEM) (Hitachi High-Tech Science Corporation, Tokyo, Japan) at 200 kV, with a magnification of 50000x.

Tetraspanin-positive extracellular vesicles identification using a bead-based flow cytometry assay

Capture beads conjugated with CD9, CD63, and CD81 antibodies from the MACSPlex Exosome Kit, Human (Miltenyi Biotec GmbH, Bergisch Gladbach, Germany, catalog no. 130-122-209) were used to bind tetraspanin-positive EVs. After capture, bead-associated EVs were labeled with allophycocyanin (APC), a fluorescently conjugated EV affinity reagent. The difference in APC median fluorescence intensity (MFI) was acquired using a BD FACSAria III flow cytometer (Becton Dickinson, Franklin Lakes, NJ, USA) and FACS Diva v8.01 (Becton Dickinson, Franklin Lakes, NJ, USA). Data analysis was performed with FlowJo v10.2 (BD Biosciences, Ashland, OR, USA).

Angiopoietin-2 (ANGPT2) and cell migration-inducing protein (CEMIP) analysis by western blot

Western blot analysis was subsequently conducted on EV suspensions isolated via DGU to identify and quantify EV ANGPT2 and CEMIP. The EV suspensions were lysed using an equal volume of ice-cold RIPA buffer (Abcam, Cambridge, UK, catalog no. ab156034) supplemented with Protease Inhibitor Mix M (SERVA Electrophoresis GmbH, Heidelberg, Germany, catalog no. 39102.01). The lysate was then subjected to a single centrifugation at 14,000 x g for 15 minutes at 4˚C. The protein concentration in the resulting supernatant was determined using a method developed by Iwata and Nishikaze, which involves the reaction of proteins with benzethonium chloride in a basic medium [29]. The mean protein concentration was 250.35 μg/100 μl (ranging from 165.44 μg/100 μl to 275.9 μg/100 μl) for LLC patients, 193.86 μg/100 μl (ranging from 160.89 μg/100 μl to 210.08 μg/100 μl) for LCM patients, and 176.51 μg/100 μl (ranging from 166.00 μg/100 μl to 196.73 μg/100 μl) for HC. The supernatant was combined with an equal volume of 2× Laemmli Sample Buffer (Bio-Rad Laboratories, Hercules, CA, USA, catalog no. 1610737) and 5% β-mercaptoethanol (Bio-Rad Laboratories, catalog no. 1610710), followed by denaturation at 95°C for five minutes.

An input of 20 μg of protein was loaded in wells from 10% Mini-PROTEAN® TGX Stain-Free™ Protein Gels (Bio-Rad Laboratories, catalog no. 4568034, 10 well, 50 µL) for protein electrophoresis with Tris/Glycine/SDS Running Buffer (Bio-Rad Laboratories, catalog no. 1610732). EV proteins were separated by sodium dodecyl sulfate-polyacrylamide gel electrophoresis (SDS-PAGE) in the Mini-PROTEAN® Tetra Vertical Electrophoresis Cell System (Bio-Rad Laboratories, Hercules, CA, USA).

The proteins were transferred onto polyvinylidene difluoride (PVDF) membranes using the Trans-Blot® Turbo™ Transfer Pack (Bio-Rad Laboratories, catalog no. 1704156) and the Trans-Blot® Turbo™ Transfer System (Bio-Rad Laboratories, Hercules, CA, USA). In order to reduce non-specific binding, the membranes were incubated with a blocking buffer containing TBST (tris-buffered saline (TBS) + 0.1% Tween® 20) and 3% bovine serum albumin (BSA). EV-derived ANGPT2 was identified after overnight incubation at 4°C with a primary rabbit anti-human anti-angiopoietin 2 (ANGPT2)/ANG2 antibody (EPR2891(2)), catalog no. ab155106, at a dilution of 1:1000. EV-derived CEMIP was identified after overnight incubation at 4°C with a primary rabbit anti-human CEMIP/KIAA1199 (KIAA1199 Polyclonal Antibody, Invitrogen™, Thermo Fisher Scientific Inc., Waltham, MA, USA, catalog no. PIPA5106288), at a dilution of 1:1000. The incubation with peroxidase-conjugated secondary goat anti-rabbit antibody (Invitrogen Goat anti-Rabbit IgG (H+L) Cross-Adsorbed Secondary Antibody, HRP, Thermo Fisher Scientific Inc., Waltham, MA, USA, catalog no. G-21234) was performed for one hour, at room temperature, at a dilution of 1:5,000. Chemiluminescent detection was achieved using the Clarity™ Western ECL Substrate kit (Bio-Rad Laboratories, catalog no. 1705061), and the images were captured and analyzed for band intensity using the ChemiDoc XRS+ System (Bio-Rad Laboratories, Hercules, CA, USA) and ImageLab™ software version 6.1.0 (Bio-Rad Laboratories, Hercules, CA, USA). The normalization method for the quantification of ANGPT2 and CEMIP from EVs used total EV protein content as a reference, ensuring accurate normalization regardless of EV purity. Meanwhile, this type of normalization avoided the problem with variable HKP expression in different pathological states. Around 20 µg of protein per lane has been loaded into the SDS-PAGE gel. Before immunoblotting, stain-free gel imaging to measure total lane protein intensity was performed using ImageLab™ software. This confirmed equal protein loading before transfer. Next, imaging was performed on the membranes before blocking to capture the total protein signal using the stain-free imaging mode, allowing for the measurement of total protein intensity and verification of transfer efficiency. After chemiluminescent detection, we quantified our protein of interest and normalized it to the total protein intensity per lane from the pre-blocking membrane image.

Systemic immune inflammation index calculation

From the complete blood counts of each patient (using preoperative values for those in the two oncological groups), we extracted routine laboratory variables, including platelets (P), neutrophils (N), and lymphocytes (L), and calculated the SII index using the formula: SII=P × N / L. The average SII value for each group was determined by summing the SII values of all patients in that group and dividing by the total number of patients.

Statistical analysis

The statistical analysis comprised both descriptive (mean, median, and standard deviation (SD)) and inferential approaches. Categorical variables were evaluated using the chi-square test and Fisher's exact test. For quantitative variables with a normal distribution, results were expressed as mean ± SD, and comparisons were made using the student's t-test. In cases where the quantitative data did not follow a normal distribution (reported as median and range), the Mann-Whitney U test was applied. The Mantel-Cox test was utilized to calculate p-values, while correlations were assessed using Spearman's rank correlation test. A significant level of p < 0.05 was considered, with a 95% confidence interval. Statistical analyses were conducted using GraphPad Prism version 8 (GraphPad Software, San Diego, CA, USA) and IBM SPSS Statistics for Windows, Version 27 (Released 2020; IBM Corp., Armonk, New York, United States).

## Results

Demographic and clinical data

The study included three groups of patients. The first group consisted of 11 patients with localized lung cancer (LLC group) and no evidence of metastases. Among them, 72.7% (n=8) were male (p=0.621). All cases were histologically confirmed as lung adenocarcinoma. Most patients had a history of smoking, and the majority of tumors were located in the middle pulmonary lobe, with an average diameter of 42.01 mm. The average value of the SII index in this group was 999.46. Gross total resection was performed in all patients.

The second group included 13 patients with primary lung cancer and one or more brain metastases (LCM group), all histologically confirmed as originating from lung cancer. Among them, 53.8% (n=7) were male (p=0.621), and 92.3% (n=12) had a history of smoking. Most patients in this group were diagnosed with adenocarcinoma, while two were diagnosed with SCLC. These two SCLC cases underwent neurosurgical treatment due to the absence of a histological diagnosis and the presence of large, single, accessible lesions located in the posterior fossa, which caused a mass effect. The average diameter of the primary lung tumors in this group was approximately 54.1 mm, about 10 mm larger than those in the first group, with the superior pulmonary lobe being the most frequently involved. No statistically significant differences were observed in tumor distribution between the right and left lungs in the two groups. Regarding brain metastases, the majority were located in the frontal lobe, followed by the cerebellum, with an average diameter of 54.1 mm. The average SII index value in this group was 1349.42. Gross total resection of the cerebral lesions was performed in all patients in this group.

The third group consisted of 12 healthy subjects (HC group), of whom 83.3% (n=10) had a smoking history and 58.3% (n=7) were male (p=0.621), with an average age of 59 years. The average SII value in the healthy group was 505.43.

Table 1 provides a detailed summary of the demographic, histological, imaging, and inflammatory aspects of the three groups of subjects included in this study.

**Table 1:** Demographic, histopathological, imaging, and inflammatory characteristics of the three groups of patients SCLC: small cell lung cancer; LLC: localized lung cancer; LCM: lung cancer with brain metastases; HC: healthy controls; KPS: Karnofsky performance score; SD: standard deviation; confidence interval: 95%; n: numbers; SII: systemic immune inflammation index; statistical significance was considered for p < 0.05; highly significant results were defined as p < 0.001

Characteristics	LLC group	LCM group	HC group	p-value	Statistical test
KPS	≥ 80	≥ 80	≥ 80	-	-
Number of patients	11	13	12	-	-
Male patients – n (%)	8 (72.7%)	7 (53.8%)	7 (58.3%)	0.621	Chi-square value (X²)=0.95
Age (years) – mean ± SD	64.82 ± 5.93	63.69 ± 8.32	59 ± 12.13	0.023	ANOVA (F-test) F-statistic=4.25
Smoking history present	9 (81.8%)	12 (92.3%)	10 (83.3%)	0.717	Chi-square test (X²)=0.664
Adenocarcinoma – n (%)	11 (100%)	11 (84.6%)	-	0.537	Chi-square test (X²)=0.381
SCLC – n (%)	0 (0%)	2 (15.4%)	-	0.537	Chi-square test (X²)=0.381
SII mean ± SD	999.46 ± 345.32	1349.42 ± 999.13	505.43 ± 227.85	0.0006	Wilcoxon-Mann-Whitney test LLC vs. HC U value=122.0
				0.028	Wilcoxon-Mann-Whitney test LCM vs. HC U value=119.0
Average diameter of the primary lung tumor (mm) – mean ± SD	42.01 ± 19.5	54.1 ± 28.14	-	0.862	Wilcoxon-Mann-Whitney test U value=68.0
Superior pulmonary lobe location – n (%)	4 (36.4%)	6 (46.2%)	-	0.697	Fisher exact test
Middle pulmonary lobe location – n (%)	6 (54.5%)	3 (23.1%)	-	0.206	Fisher exact test
Inferior pulmonary lobe location – n (%)	3 (27.3%)	2 (15.4%)	-	0.630	Fisher exact test
Average diameter of brain metastases (mm) – mean ± SD	-	54.1 ± 26.7	-	-	-
Frontal lobe location – n (%)	-	9 (69.23%)	-	-	-
Parietal lobe location – n (%)	-	4 (30.77%)	-	-	-
Temporal lobe location – n (%)	-	2 (15.38%)	-	-	-
Occipital lobe location – n (%)	-	1 (7.69%)	-	-	-
Cerebellar location – n (%)	-	5 (38.46%)	-	-	-

Quantifiable results of extracellular vesicle-associated angiopoietin-2 and extracellular vesicle cell migration-inducing protein 

**Figure 1:**
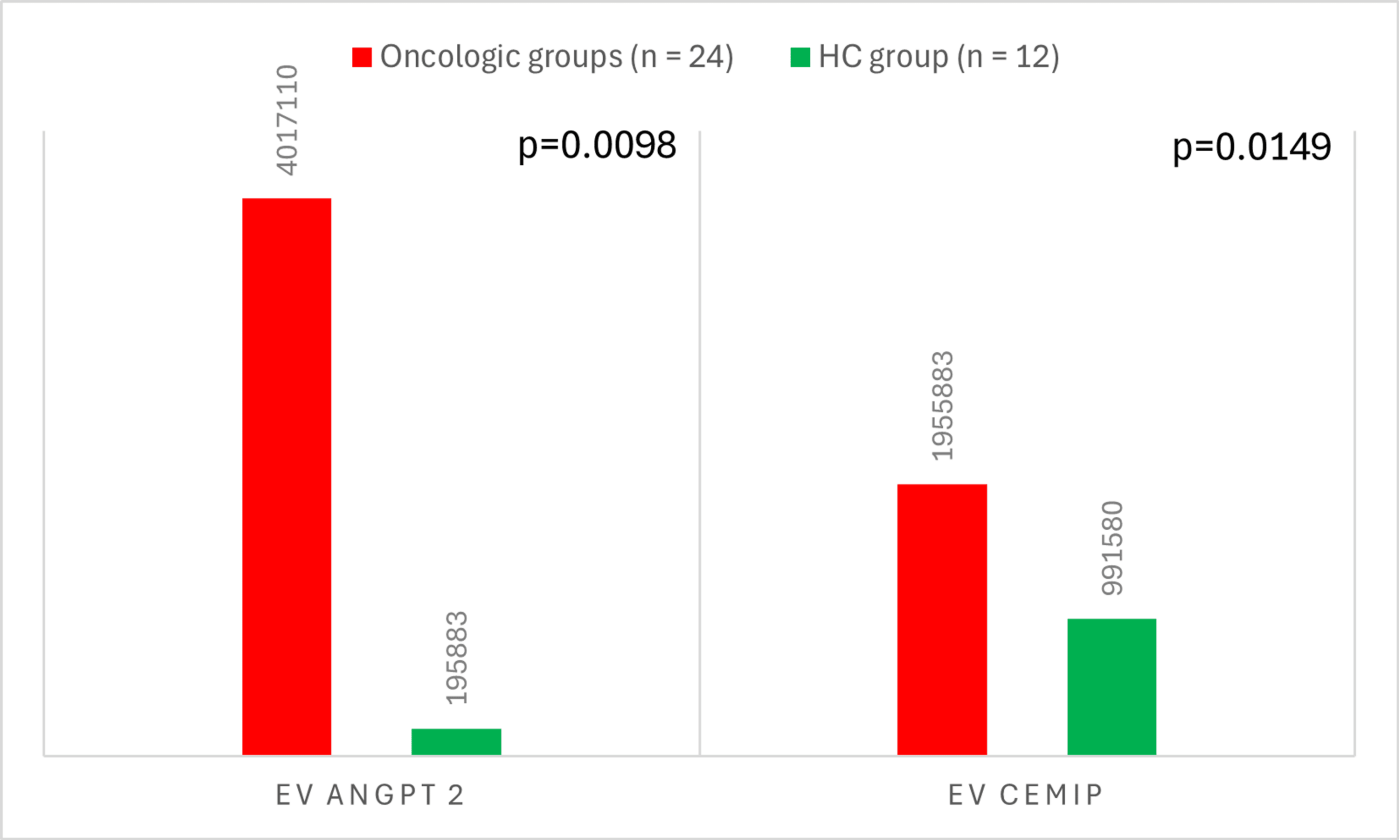
Expression levels of EV ANGPT2 and EV CEMIP in oncologic (LLC+LCM groups) and healthy control groups Y-axis: mean expression; X-axis: exosomal proteins (EV ANGPT2, EV CEMIP); bars: red (oncologic group)-patients diagnosed with cancer; green (healthy controls (HC))-individuals without cancer EV: extracellular vesicle; ANGPT2: angiopoietin-2; CEMIP: cell migration-inducing protein; LLC: localized lung cancer; LCM: lung cancer with brain metastases

Table 2 and Figure 1 show a comparison of the protein levels measured by western blot between oncologic patients (LLC and LCM groups) and healthy controls. Preoperative EV ANGPT2 levels were significantly higher (p < 0.001) in oncologic patients (4017110.67) compared to healthy controls (195883.46). A similar trend was observed for EV CEMIP levels, with significantly higher values (p < 0.05) in oncologic patients (1955883.46) compared to healthy individuals (991580.42).

**Table 2:** Quantifiable results of the western blot images which were captured and analyzed for band intensity This was done using the ChemiDoc XRS+ System (Bio-Rad Laboratories, Hercules, CA, USA) and ImageLab™ software version 6.1.0 (Bio-Rad Laboratories, Hercules, CA, USA). Comparison of the average EV ANGPT2 and CEMIP values between the combined oncologic groups (LLC group + LCM group) and healthy subjects. Statistical test used: Wilcoxon Mann-Whitney test (CI 95%); *this represents the average preoperative EV ANGPT2 and CEMIP values of the patients included in the oncologic groups; statistical significance was considered for p < 0.05; highly significant results were defined as p < 0.001. EV: extracellular vesicle; ANGPT2: angiopoietin-2; CEMIP: cell migration-inducing protein; LLC: localized lung cancer; LCM: lung cancer with brain metastases

Levels	Oncologic groups (n=24)	HC group (n=12)	p-value	Statistical test used
EV ANGPT 2 – Mean ± SD	*4017110.67 ± 5194997.5	195883.46 ± 1280938.1	0.0098	Wilcoxon-Mann-Whitney test U-statistic=216.0
EV CEMIP – Mean ± SD	*1955883.46 ± 1280938.1	991580.42 ± 788219.62	0.0149	Wilcoxon-Mann-Whitney test U-statistic=216.0

**Figure 2:**
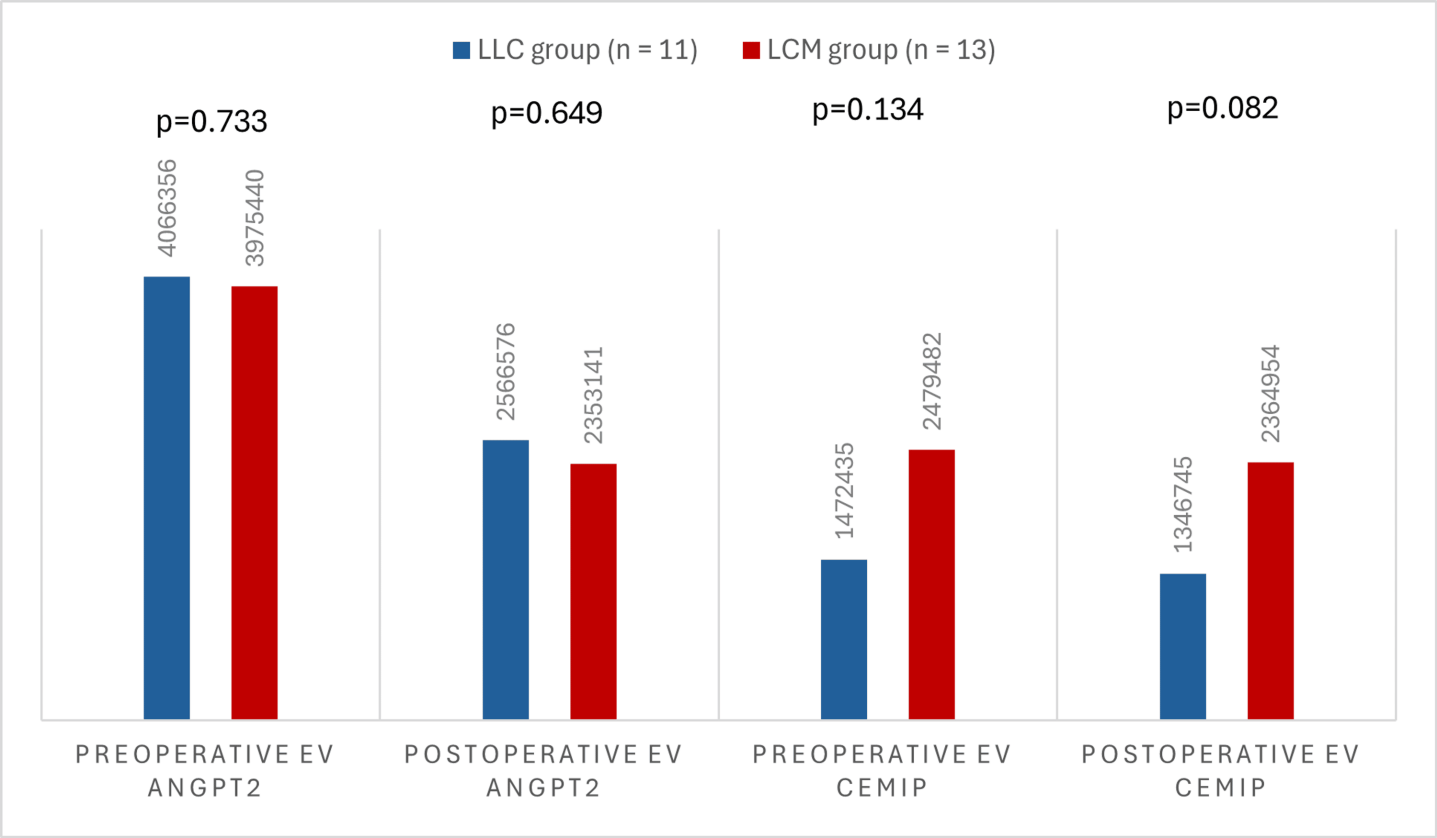
Comparison of reoperative and postoperative EV ANGPT2 and EV CEMIP levels between LLC and LCM groups Y-axis: mean expression of EV ANGPT2 and EV CEMIP; X-axis: preoperative EV ANGPT2, postoperative EV ANGPT2, preoperative EV CEMIP, postoperative EV CEMIP; bars: blue (LLC group, n=11)-patients with localised lung cancer; red (LCM group, n=13)-patients with lung cancer and brain metastases EV: extracellular vesicle; ANGPT2: angiopoietin-2; CEMIP: cell migration-inducing protein; LLC: localized lung cancer; LCM: lung cancer with brain metastases

The evolution of average EV ANGPT2 and CEMIP levels after surgical resection between the LLC and LCM groups is shown in Table 3 and Figure 2.

**Table 3:** Quantifiable results of the western blot images which were captured and analyzed for band intensity This was done using the ChemiDoc XRS+ System (Bio-Rad Laboratories, Hercules, CA, USA) and ImageLab™ software version 6.1.0 (Bio-Rad Laboratories, Hercules, CA, USA). Comparison of the preoperative and postoperative average EV ANGPT2 and CEMIP values between LLC group and LCM group. The statistical test used was the Mann-Whitney test (CI 95%); statistical significance was considered for p < 0.05; highly significant results were defined as p < 0.001. EV: extracellular vesicle; ANGPT2: angiopoietin-2; CEMIP: cell migration-inducing protein; LLC: localized lung cancer; LCM: lung cancer with brain metastases

Level	LLC group (n=11)	LCM group (n=13)	p-value	Statistical test used
Preoperative EV ANGPT2 – Mean ± SD	4066356.90 ± 3954297.72	3975440.77 ± 6220329.69	0.733	Wilcoxon-Mann-Whitney test U value=60.5
Postoperative EV ANGPT2 – Mean ± SD	2566576.64 ± 2286091.76	2353141 ± 2417967.24	0.649	Wilcoxon-Mann-Whitney test U value=60.5
Preoperative EV CEMIP – Mean ± SD	1472435.55 ± 1100853.48	2479482.31 ± 1931257.63	0.134	Wilcoxon-Mann-Whitney test U value=60.5
Postoperative EV CEMIP – Mean ± SD	1346745.27 ± 1277816.36	2364954.77 ± 1318879.557	0.082	Wilcoxon-Mann-Whitney test U value=60.5

Following surgical intervention, a decrease in the average EV ANGPT2 levels was observed in both the LLC and LCM groups. In the LLC group, the EV ANGPT2 levels decreased from a preoperative average of 4066356.90 to a postoperative average of 2566576.64. Similarly, in the LCM group, the preoperative average EV ANGPT2 level decreased from 3975440 to 2353141 postoperatively. However, this trend was not consistent for all patients. Notably, five patients from the LLC group and five from the LCM group presented higher postoperative EV ANGPT2 values.

The difference between preoperative and postoperative average EV CEMIP levels was not significant in either group. In the LLC group, the preoperative average was 1472435.55, compared to a postoperative average of 1346745.27. Similarly, in the LCM group, the postoperative average (2364954.77) was slightly lower than the preoperative value (2479482.31). However, a notable difference was observed when comparing preoperative EV CEMIP levels between the two groups. Patients in the metastatic stage (LCM group) had much higher values (2479482.31) compared to those with localized lung cancer (LLC group), whose average value was 1472435.55 (p=0.134).

Isolated extracellular vesicles were identified by scanning and transmission electron microscopy

**Figure 3:**
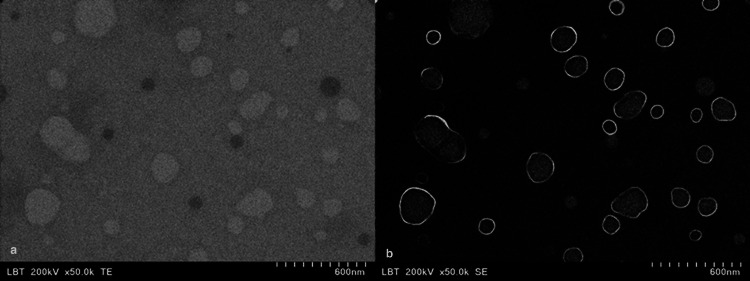
Scanning and transmission electron microscopy (STEM) findings a: TEM image acquisition; b: SEM image acquisition TEM: transmission electron microscopy; SEM: scanning electron microscopy

The EV suspensions obtained by density gradient ultracentrifugation (DGU) were characterized by STEM. The nanoscale particles appeared small and rounded, with sizes ranging in the interval of 30-1000 nm. The EVs showed an outer layer with varying thickness and higher contrast compared to the inner content, as shown in Figure 3.

Extracellular vesicles characterization by flow cytometry

**Figure 4:**
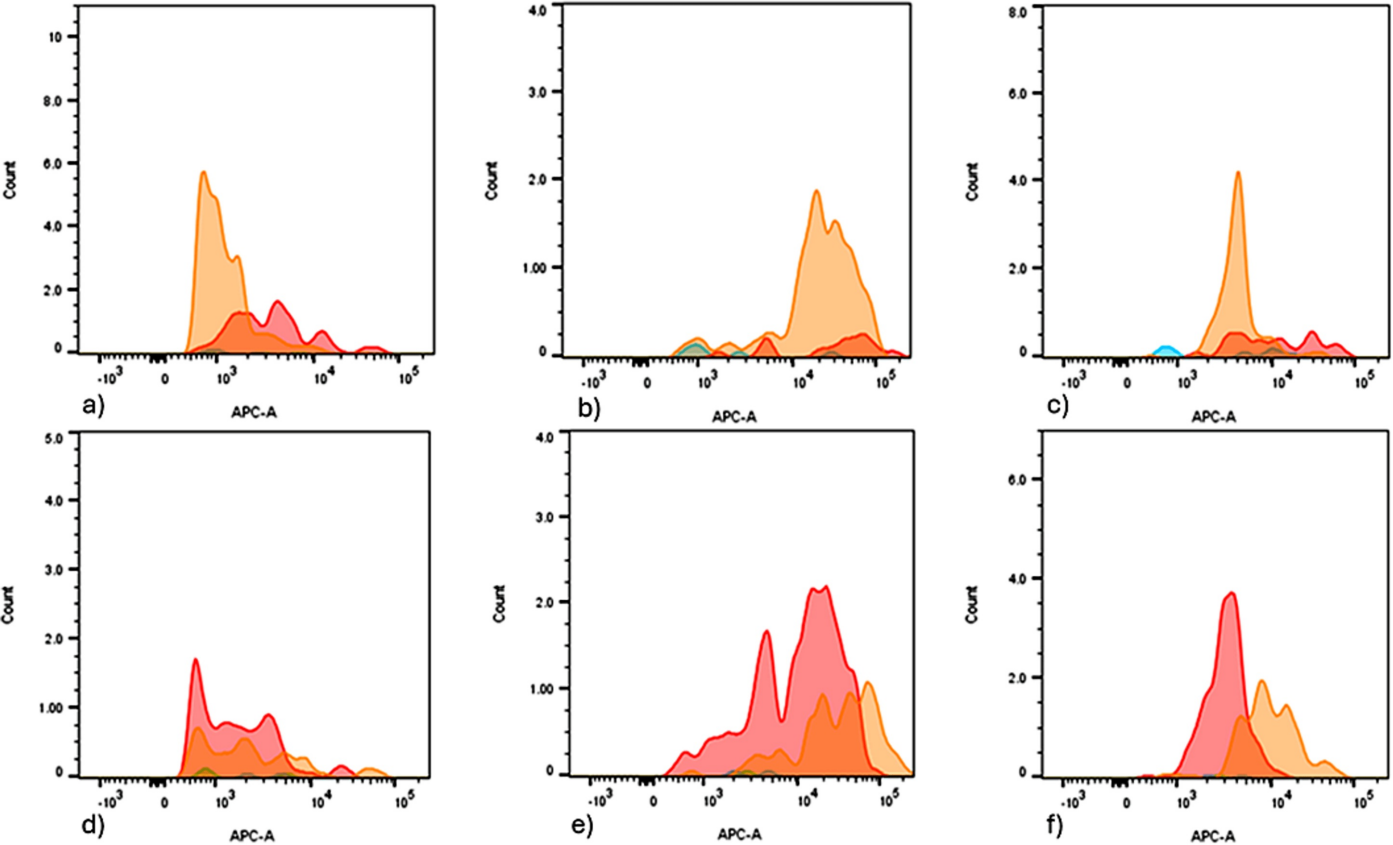
Histograms of two patients illustrating variations in APC MFI for EVs positive for CD9, CD63, and CD81 in different conditions LLC patient: (a) CD9, (b) CD63, and (c) CD81; LCM patient: (d) CD9, (e) CD63, and (f) CD81; EVs analyzed under buffer-only control (green), isotype control (blue), patient pre-operative (red), and post-operative (orange) conditions. APC: allophycocyanin; MFI: median fluorescence intensity; EV: extracellular vesicle

EV suspensions isolated from plasma were used to identify tetraspanin-positive EVs using a bead-based flow cytometry method. The difference in APC MFI from singlet-gated beads reflected variations in EV concentration, epitope density, and relative abundance of EV subsets defined by the presence of tetraspanins. Figure 4 illustrates the variations in APC MFI between the buffer-only control, isotype-conjugated capture bead control, and tetraspanin-positive EVs after bead incubation with either buffer alone or EV suspensions obtained from the plasma of LLC and LCM patients. Histograms are exported from the FlowJo v.10.2 Image Layout Editor.

The analysis of tetraspanin-positive EVs showed differences in APC MFI between LLC and LCM patients, indicating distinct tumor biology that affects the release and composition of EVs.

Angiopoietin-2 (ANGPT2) and cell migration-inducing protein (CEMIP) profile in extracellular vesicle cargo from lung cancer patients with and without brain metastasis

**Figure 5:**
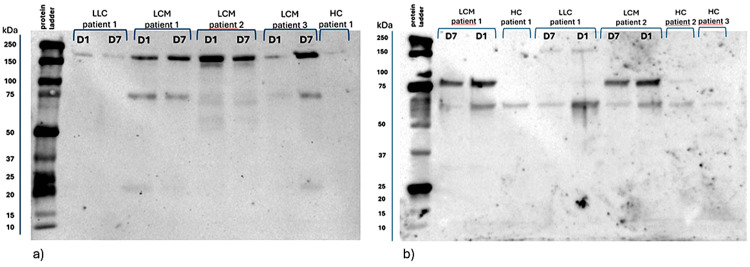
Western blot findings a: Representative western blots corresponding to EV ANGPT2 at molecular weights of approximately 150-250 kDa representing post-translational modifications and isoforms. Additionally, a faint band between 50 and 75 kDa was intermittently detected, showing lower intensities; b: Representative western blots corresponding to EV CEMIP at molecular weights of approximately 75-100 kDa, which may represent either proteolytic fragments of the mature 150 kDa CEMIP molecule or unidentified splice variants of CEMIP. Additionally, a faint band between 50 and 75 kDa can be observed, showing lower intensities. Some of the patients included in the oncologic groups presented protein bands at the expected molecular weight of 150 kDa, as can be observed in patient 1 from the LLC group. D1: protein bands resulted from preoperative blood samples; D7: protein bands resulted from postoperative blood samples; kDa: kilodaltons; EV: extracellular vesicle; ANGPT2: angiopoietin-2; CEMIP: cell migration-inducing protein; LLC: localized lung cancer; LCM: lung cancer with brain metastases

Following western blot analysis, ANGPT2 and CEMIP band intensities were quantified on PVDF membranes after incubation with specific antibodies and chemiluminescent detection. When the membranes were probed with the anti-ANGPT2 antibody, a distinct band between 150 and 250 kilodaltons (kDa) was consistently observed in the samples of cancer patients, with variable intensities, suggesting a potential upregulation of ANGPT2 oligomerization in the EV cargo. Additionally, a faint band between 50 and 75 kDa was intermittently detected, showing lower intensities. ANGPT2 is predicted to have a molecular mass of 55-60 kDa, depending on its post-translational modifications and isoforms (https://www.uniprot.org/uniprotkb/O15123/entry). When the membranes were probed with the anti-CEMIP antibody, in some patients included in the oncologic groups, faint bands at the expected molecular weight of 150 kDa were seen. More prominent bands with variable intensities between 75 and 100 kilodaltons (kDa) were consistently observed in the samples from cancer patients. The 75-100 kDa species may represent either proteolytic fragments of the mature 150 kDa CEMIP molecule or unidentified splice variants of CEMIP. Additionally, a faint band between 50 and 75 kDa was intermittently detected, showing lower intensities. CEMIP is predicted to have a molecular mass of approximately 150 kDa, depending on its post-translational modifications and isoforms (https://www.uniprot.org/uniprotkb/Q8WUJ3/entry). Figure 5a shows typical western blots that show EV ANGPT2 protein bands from blood samples taken before and after surgery from one patient in the LLC group, three patients in the LCM group, and one healthy patient. The blot emphasizes the differences in ANGPT2 expression among these groups. We can observe that in patients 1 and 3 from the LCM group, postoperative ANGPT2 expression is increased after surgical resection of the brain metastases, but in patient 1 from the LLC group, ANGPT2 expression decreases after complete resection of the lung tumor. Figure 5b displays the representative western blots revealing EV CEMIP protein bands from postoperative and preoperative blood samples of two patients from the LCM group, one patient from the LLC group, and three healthy patients. Here, we can observe a decrease in postoperative CEMIP expression in all three cancer patients.

## Discussion

Angiogenesis is essential for lung cancer growth, progression, and metastasis [30,31]. This biological process, responsible for the formation of new blood vessels, is controlled by an uneven ratio of pro-angiogenic and anti-angiogenic factors. The angiopoietin family, consisting of ANGPT1, ANGPT2, ANGPT3 (mouse ortholog), and ANGPT4, interacts with the endothelial cell-specific receptor tyrosine kinase Tie-2 to regulate vascular remodeling and function. ANGPT2, a proangiogenic factor with minimal expression in normal tissues and elevated levels in tumor endothelial cells, interacts with VEGF and other factors to drive tumor angiogenesis and inflammation [18,32]. As an extracellular protein and key ligand of the Tie2 receptor, ANGPT2 plays a crucial role in regulating these processes. It is released from endothelial cells via exosomes, a process inhibited by the PI3K/Akt/eNOS signaling pathway but promoted by the syndecan-4/syntenin pathway [33-35].

Elevated CEMIP expression is associated with a bad prognosis in NSCLC, particularly in metastatic cases, where it promotes EMT and enhances tumor invasiveness. Additionally, CEMIP contributes to angiogenesis by stimulating VEGF signaling and facilitates a pro-inflammatory tumor microenvironment, further supporting tumor growth and metastasis [24]. Recent evidence suggests that CEMIP is also involved in extracellular vesicle-mediated communication, where it may influence metastatic spread by modifying the tumoral niche [25].

One of our goals for this study was to identify the presence of ANGPT2 and CEMIP in the EV cargo from the peripheral blood of patients with LC with or without brain metastases. Although previous research has documented ANGPT2 and CEMIP in EVs across different tumor types [33-36], to our knowledge, no previous study has evaluated the presence of ANGPT2 and CEMIP in EVs of lung cancer patients. Based on western blot analysis, STEM, and flow cytometric analysis, we have confirmed the presence of ANGPT2 and CEMIP within the EV cargo, supporting their potential relevance in lung cancer progression.

Another goal was to quantify the differences in preoperative average EV ANGPT2 and CEMIP levels between the LLC and LCM groups. We observed relatively similar preoperative average EV ANGPT2 levels between patients with localized lung cancer and those with lung cancer and brain metastases, but on the other hand, the preoperative average EV CEMIP levels were 59% higher in the LCM group compared with the LLC group.

Subsequently, we analyzed how average EV ANGPT2 levels and average EV CEMIP levels changed following gross total surgical resection. Our results indicate that, on average, seven days following the surgical intervention, the average EV ANGPT2 levels decreased by 36% in the LLC group and by approximately 40% in the LCM group. However, these results were not statistically significant and were not seen in each patient because five patients from the LCM group and five patients from the LLC group exhibited significantly higher postoperative EV ANGPT2 levels seven days after gross total resection compared to their preoperative levels. Kopczynska et al. showed that surgical resection of stage I NSCLC induces changes in plasma angiogenic factors, particularly an increase in ANGPT2 and VEGF levels by postoperative day seven, followed by a decrease by day 30 [37]. Ng et al. showed that surgical resection for early-stage NSCLC induces significant pro-angiogenic changes, particularly marked by increased ANGPT2 levels and decreased ANGPT1 levels postoperatively [38]. Zhou et al. concluded that plasma ANGPT2 levels are significantly increased in NSCLC patients compared to healthy subjects and increase further following surgery, peaking around postoperative day 14 and remaining elevated for at least eight weeks. These elevated levels enhance angiogenesis and endothelial proliferation, suggesting a potential role in postoperative cancer recurrence. He concluded that targeting ANGPT2 through anti-ANGPT2 therapy could be a promising strategy for adjuvant treatment in NSCLC [39].

Our findings indicate that the average EV CEMIP values were insignificantly affected by gross total surgical resection, with the postoperative average EV CEMIP values being slightly less than the preoperative ones in both oncologic groups. Currently, there is limited evidence regarding the changes in CEMIP levels following surgical resection in lung cancer. However, studies in other cancers suggest that CEMIP expression may correlate with tumor presence and progression. For instance, in hepatocellular carcinoma, high CEMIP expression in surgically resected specimens was associated with a higher risk of lung metastasis, indicating that elevated CEMIP levels may persist post-surgery in aggressive cancer types [40].

We have also compared the preoperative average EV ANGPT2 and CEMIP levels between cancer patients and healthy subjects, revealing statistically significant differences. We observed that the preoperative average EV ANGPT2 levels in the oncologic groups were 20.5 times higher compared to the control group (p=0.009). These findings align with previous studies reporting elevated serum ANGPT2 levels and elevated circulating ANGPT2 messenger RNA in NSCLC and their association with disease progression and poor survival outcomes [41-43]. Park et al. reported a strong association between serum levels of ANGPT2 and survival, showing that elevated serum levels of ANGPT2 are correlated with shorter survival [44]. A recent meta-analysis has revealed that ANGPT2 serum levels increase gradually according to the cancer stage, with grade 4 patients having the highest serum values [21]. Contrary to the results from the abovementioned studies, our results have shown slightly elevated EV ANGPT2 average levels in patients from the LLC group compared to the LCM group, but the differences were not statistically significant. This may be attributed to the small number of patients included in our study, which could have influenced the statistical power of the analysis.

Notably, the average EV CEMIP values were nearly twice as high in the oncologic groups compared to the healthy group (p=0.01), suggesting its involvement in tumor development. Recent studies indicate that CEMIP also plays a role in metabolic reprogramming, promoting tumor adaptation to hypoxic environments. Additionally, CEMIP has been implicated in resistance to apoptosis, which may contribute to tumor persistence even after treatment [24].

The oligomerization domain of ANGPT2 is crucial for vascular remodeling and metastatic dissemination. Structural modifications within this domain may impact tumor progression and facilitate the dissemination of cancer cells to the lungs [45]. Our western blot images highlighted a personalized profile of patients. The postoperative EV ANGPT2 levels are variable compared to preoperative results, regardless of whether the patient presents with localized cancer or in the metastatic stage. Moreover, ANGPT2 western blot analysis consistently revealed a prominent protein band in the 150-250 kDa range in cancer patient samples and some samples of non-cancerous subjects, suggesting an upregulation of ANGPT2 oligomerization in the EVs. This observation aligns with the understanding that ANGPT2, which typically has a predicted molecular mass of 55-60 kDa, can undergo post-translational modifications and oligomerization, leading to the formation of larger molecular complexes in the case of ANGPT2. The detection of a faint band in the 50-75 kDa range further highlights the variability in ANGPT2's structural states, likely reflecting its different isoforms or levels of modification. The oligomerization state of ANGPT2 is particularly significant as it directly influences its biological activity. ANGPT2 oligomerization affects its binding affinity and interaction with the Tie2 receptor, a critical modulator of endothelial cell functions. These findings suggest that the altered oligomeric state of ANGPT2 in cancer-related EVs may play a role in tumor angiogenesis and progression by modulating Tie2-mediated signaling pathways [46]. As Kim et al. showed, the oligomerization of ANGPT2 may be necessary for consistent Tie2 activation by promoting its clustering, while improper oligomerization may inhibit Tie2 activation by preventing further binding of correctly oligomerized ANGPT2 and ANGPT1. Although SDS-PAGE analysis previously performed suggests that ANGPT2 forms disulfide-linked dimers, rotary metal-shadowing transmission electron microscopy analysis conducted by Kim et al. indicates the presence of trimers, tetramers, and higher-order multimers. Thus, endogenous ANGPT2 likely exists in multiple oligomeric forms [47]. Another research group investigated the role of the amino-terminal oligomerization domain of ANGPT2 in lung metastasis in mice. The study focuses on a naturally occurring isoform of ANGPT2 with lower oligomerization. This ANGPT2 oligomer variant impairs venous development, affects primary tumor growth and vascularization, and promotes lung metastasis by destabilizing pulmonary vasculature. The authors also showed that, in vitro, ANGPT2443 in its monomeric form inhibited Tie-2 activation [45].

On the other hand, CEMIP western blot analysis revealed some faint bands at the expected molecular weight of 153 kDa in some of the cancer patients and more prominent protein bands in the 75-100 kDa range, respectively, in the 50-75 kDa range in most cancer patients. These protein bands located at lower molecular weights than expected may represent either proteolytic fragments of the mature 150 kDa CEMIP molecule or alternative splice variants of CEMIP [48]. The lower molecular weight bands observed in our study could be explained by the reduction of disulfide bonds under reducing conditions. The 50-100 kDa variants could represent a proteolytically cleaved form of CEMIP, where the protein is processed into smaller fragments under the reducing conditions, possibly due to cleavage events that occur during sample preparation or in vivo.

CEMIP could exist as multiple isoforms with differing molecular weights, possibly due to alternative splicing or truncation, and one of these isoforms might migrate at 50-100 kDa when the protein is denatured and reduced. Further proteomic studies must be conducted to validate potential CEMIP isoforms and their functional roles in cancer.

Larger tumor size is generally associated with higher TNM stage, increased invasiveness, and worse prognosis. Smaller tumors (<3 cm) are generally less aggressive, have lower metastatic potential, and are often diagnosed at an earlier stage, whereas larger tumors (>5 cm) have a higher likelihood of local invasion, greater metastatic potential, poorer differentiation, and increased recurrence rates after surgical resection. In NSCLC, tumor size is a well-established prognostic factor, with larger tumors correlating with lower overall survival and disease-free survival [49-51]. Our results showed that the average diameter of lung tumors in the LCM group was more than 5 cm, being, on average, 12.09 mm larger compared to the LLC group. These findings reinforce the notion that larger tumor size is associated with more aggressive disease and a higher risk of metastasis.

In NSCLC, tumor localization shows a predilection for the upper pulmonary lobes. This distribution is influenced by factors such as prolonged exposure to inhaled carcinogens, lower ventilation and clearance in the upper lobes, and differences in lung perfusion, with lower lobes having better blood supply and higher oxygenation [52,53]. Our results revealed an upper pulmonary lobe predilection as well. In the LLC group, 54.5% (n=6) of the tumors were located in the middle pulmonary lobe, while in the metastatic group, 46.2% (n=6) of the tumors were found in the superior pulmonary lobe.

Studies have revealed a complex correlation between the location of brain metastases and lung cancer progression, which is influenced by multiple factors like tumor histology, molecular profile, and metastatic pathways. NSCLC metastases have a predilection for the frontal and parietal lobes, likely due to the high vascularization of these regions. On the other hand, SCLC metastases are more frequently found in the cerebellum, leading to more severe neurological symptoms and poorer outcomes [54,55]. Our results reveal a higher predilection of adenocarcinoma metastases for the frontal lobes and cerebellum, followed by the parietal lobes. Both of our patients with SCLC presented with cerebellar metastases.

The systemic immune-inflammation index has emerged as a valuable prognostic and predictive biomarker in lung cancer, reflecting the balance between systemic inflammation and immune suppression. Elevated SII levels are consistently associated with more aggressive tumor behavior, advanced disease stage, and poorer overall survival and progression-free survival in both NSCLC and SCLC patients. Studies have shown that high SII correlates with increased tumor size, lymph node involvement, and metastatic potential, as well as reduced responses to immunotherapy and chemotherapy. Given its accessibility through routine blood tests, SII serves as a practical and cost-effective tool for risk stratification, guiding treatment decisions, and improving personalized lung cancer management [56,57]. H. Li et al. reported that in brain metastases from lung adenocarcinoma, an SII of ≤ 1218.81 correlated with improved survival, particularly in patients with EGFR wild-type, where it showed significant prognostic value in both univariate and multivariate analyses [58]. Hu et al. concluded in his study on hepatocellular carcinoma that SII ≥ 330 is a strong prognostic marker for poor overall survival and relapse-free survival, being strongly correlated with early recurrence, large tumors, vascular invasion, and elevated circulating tumor cell levels, emphasizing its potential as a valuable tool for optimizing treatment strategies [59]. Our results showed a higher average SII value in patients from the LCM group (1349.42) compared to the LLC group, which had an average SII value of 999.46. In contrast, healthy subjects had a significantly lower average SII value of 505.43, approximately half of that observed in cancer patients (p < 0.001). These findings, along with the elevated CEMIP expression in the EV cargo of cancer patients, underscore the heightened pro-inflammatory status associated with advanced stages of cancer.

Although our study offers valuable insights, several limitations should be acknowledged. First, the relatively small sample size may limit the generalizability of our findings, as the stringent inclusion criteria focused exclusively on patients without metastatic lesions outside the brain or lungs. Second, the absence of longitudinal data beyond the immediate postoperative period restricts our ability to assess ANGPT2 and CEMIP dynamics over time. Third, we have not yet established optimal quality control measures for western blot analysis of samples containing EVs, but the western blotting protocol has previously been used by our team from the Department of Immunology from the Advanced Medical and Pharmaceutical Research Center, affiliated with the "George Emil Palade" University of Medicine, Pharmacy, Science, and Technology in Târgu Mureș, Romania, for identifying extracellular vesicle biomarkers in other pathologies, without establishing the optimal control at this point [60,61]. Some functional studies should be conducted to validate the roles of ANGPT2 and CEMIP in lung cancer progression. In our research group, in vitro studies such as the endothelial permeability assay, where endothelial monolayers are treated with recombinant ANGPT2 to measure barrier integrity, can be performed using microfluidic cell culture equipment. Analyzing ANGPT2 and CEMIP expressions and their effect on tumor microenvironment cells through single-cell RNA sequencing could provide valuable insights into studied biomarkers' role in lung cancer progression. Additionally, a xenograft model is feasible for us, where CEMIP- or ANGPT2-modified lung cancer cells can be injected into nude mice to track tumor growth and angiogenesis. Furthermore, future studies should incorporate mass spectrometry-based proteomics to validate the presence of isoforms detected by western blotting, ensuring a more comprehensive characterization of these proteins. Future research should address study limitations by including larger cohorts, extended follow-up periods, and additional functional assays.

Proper oligomerization of ANGPT2 may be necessary for consistent Tie2 activation by promoting its clustering, while improper oligomerization may inhibit Tie2 activation by preventing further binding of correctly oligomerized ANGPT2 and ANGPT1. However, the exact oligomerization state of endogenous ANGPT2 in different tissues and conditions remains unknown. SDS-PAGE analysis suggests that recombinant ANGPT2 forms disulfide-linked dimers, while RMSTEM analysis indicates the presence of trimers, tetramers, and higher-order multimers. Thus, endogenous ANGPT2 likely exists in multiple oligomeric forms rather than just as a dimer.

## Conclusions

This study confirms the presence of ANGPT2 and CEMIP in EVs isolated from the peripheral blood of patients with both localized lung cancer and lung cancer with brain metastases. Western blot analysis highlighted a significantly higher expression of a multimeric variant of ANGPT2 in EV cargo from cancer patients compared to healthy controls. Similarly, alternative splice variants of CEMIP were found in elevated levels in the EVs of cancer patients, with a nearly 60% increase in expression in those with brain metastases compared to those with localized lung cancer. While ANGPT2 and CEMIP have been previously documented in different tumor types, the characterization of specific isoforms, multimeric variants, or splice variants remains incomplete. Further studies are needed to explore the functional impact of these EV-associated protein variants and their role in disease progression across various cancer types.
